# Cognitive and behavioral factors of quality of life in patients with somatoform disorders

**DOI:** 10.1192/j.eurpsy.2021.510

**Published:** 2021-08-13

**Authors:** I. Belokrylov, S. Semikov, A. Tkhostov, E. Rasskazova, A. Yavorovskaya

**Affiliations:** 1 Department Of Psychiatry And Medical Psychology, Peoples Friendship University of Russia (RUDN University), Moscow, Russian Federation; 2 Department Of Psychiatry And Medical Psychologi, Peoples Friendship University of Russia (RUDN University), Moscow, Russian Federation; 3 Department Of Neuro- And Pathopsychology, Faculty Of Psychology, Lomonosov Moscow State University, Moscow, Russian Federation; 4 Faculty Of Psychology, Lomonosov Moscow State University, Moscow, Russian Federation; 5 Clinical Psychology, Moscow State University, Moscow, Russian Federation

**Keywords:** somatoform disorders, factors of quality of life

## Abstract

**Introduction:**

Studies of the cognitive and behavioral factors of perpetuation and quality of life in patients with somatoform disorders are important for identifying targets for psychological interventions and risk groups (Piontek et al., 2018, Dehoust et al., 2017, Schaefer et al., 2012, Flasinski et al., 2020).

**Objectives:**

To reveal beliefs and behavior in patients with somatoform disorders associated severity of somatic complaints and poorer subjective well-being.

**Methods:**

125 patients with somatoform disorders 17-68 years old filled Screening for Somatoform Symptoms (Rief, Hiller, 2003), Cognitions About Body And Health Questionnaire (Rief et al., 1998), Scale for the Assessment of Illness Behaviour (Rief, Ihle, Pigler, 2003), and Quality of Life Enjoyment and Satisfaction Questionnairie-18 (Ritsner et al., 2005).

**Results:**

Severity of somatoform symptoms is higher in patients with catastrophization of bodily sensations, autonomic sensations, belief in their bodily weakness, somatosensory amplification, scanning for bodily symptoms, and disturbances in daily activities due to illness (r=.18-.38, p<.05). Adjusting for the severity of somatoform symptoms, subjective well-being was lower in patients with higher belief in their bodily weakness and somatosensory amplification, autonomic sensations, expression of symptoms, and changes in daily activities due to illness (r=.21-.40, p<.05).

**Conclusions:**

The results suggests that regardless of symptoms severity poorer quality of life in patients with somatoform disorders is associated with beliefs about body and body perception that could be addressed in psychotherapy.

